# Exploring the lived experiences of LGBTQIA+ young people with visible differences: Being ‘*double different*’

**DOI:** 10.1111/bjhp.70118

**Published:** 2026-11-04

**Authors:** Emma Waite, Elizabeth Jenkinson, Victoria Clarke, Ella Guest

**Affiliations:** ^1^ Centre for Appearance Research University of the West of England (UWE) Bristol UK; ^2^ University of the West of England (UWE) Bristol UK

**Keywords:** appearance‐affecting conditions, intersectionality, LGBTQ+, qualitative, youth

## Abstract

**Objectives:**

Existing research suggests young people with visible differences can experience a range of psychosocial challenges, such as low self‐esteem and increased anxiety and depression. However, young people from minoritised groups are not well represented in visible difference research. This includes LGBTQIA+ young people, who experience both mental and physical health inequalities and may be at greater risk of negative body image than their cisgender/heterosexual peers. While it can be argued that LGBTQIA+ young people with visible differences may be at greater risk of negative psychosocial outcomes compared to their cisgender/heterosexual counterparts, little research explores the unique experiences of this population. Therefore, this study aimed to explore the lived experiences of LGBTQIA+ young people (aged 15–28 years) with visible differences.

**Design:**

This study used an inductive, qualitative design. The research was underpinned by a critical realist/contextualist perspective.

**Methods:**

Data were generated using online qualitative surveys and 75 young people with a range of gender identities, sexualities, and visible differences took part. Reflexive thematic analysis was used to analyse the data.

**Results:**

One overarching theme, *The weight of multiple marginalisation*, was generated that encompassed two themes: (1) *Dual identities*, *dual stigma* and (2) *Hiding to prevent hurting*.

**Conclusions:**

Analysis suggested that LGBTQIA+ young people with visible differences face unique challenges not accounted for by generic support. However, some experienced personal growth and described feeling accepted by LGBTQIA+ people. Tailored, co‐produced interventions are needed to effectively support this population and reduce the health and social inequalities they face.

**Statement of contribution**

**What is already known on this subject?**

- Young people with visible differences can experience psychosocial challenges.
- LGBTQIA+ young people are at increased risk of negative body image and face health inequalities.
- LGBTQIA+ people are not well represented in existing visible difference research.

**What does this study add?**

- LGBTQIA+ young people with visible differences face unique challenges, largely relating to intersectional stigma.
- Tailored, co‐produced interventions are needed to support LGBTQIA+ young people with visible differences.

## INTRODUCTION

The term ‘visible difference’ refers to an altered physical appearance resulting from a condition (e.g., vitiligo), injury (e.g., burns) or medical treatment (e.g., surgical scars). Research indicates many people with a visible difference adjust and cope well, whereas others face a range of psychosocial challenges (Harcourt et al., 2025). These can include negative and stigmatising reactions from others, such as teasing/bullying, staring, and questions and comments about their visible difference (Stock et al., 2013; Tiemens et al., 2013). Such reactions have a negative impact and contribute to the heightened anxiety and depression, feelings of unattractiveness, and low self‐esteem that some people with visible differences report (Dalgard et al., 2015; Sharratt et al., 2018). Currently, most visible difference research focuses on the experiences of cisgender/heterosexual people, resulting in limited insight into the experiences of LGBTQIA+ people (Cimino et al., 2021; Schneider et al., 2018; Thornton & Harcourt, 2026).

Although research is limited, some visible differences are perhaps more prevalent among LGBTQIA+ populations. For example, compared to heterosexual men, gay and bisexual men appear more likely to develop skin cancer, which can result in appearance changes due to treatment or the cancer itself (Singer et al., 2020). There is also some evidence that lesbian and bisexual women are more likely to experience acne than heterosexual women (Agrawal et al., 2004). Additionally, gender‐affirming hormone therapy for transgender people may result in certain visible differences. For example, acne and androgenetic alopecia are common side effects of testosterone and so may develop or worsen for transgender men (Rafferty et al., 2018; Yeung et al., 2019), and transfeminine patients undergoing oestrogen hormone therapy may develop the skin condition melasma (Yeung et al., 2019). Finally, research indicates that men who have sex with men and transgender people are disproportionately affected by Human Immunodeficiency Virus (HIV; UN AIDS, 2024), and that medications to treat HIV can cause lipodystrophy (a fat redistribution condition), which can result in judgements towards one's appearance as well as underlying HIV status (Drabarek et al., 2025; Riley, 2002).

LGBTQIA+ people may also experience additional, unique psychosocial challenges compared to cisgender/heterosexual people with visible differences. For example, evidence indicates individuals with psoriasis who report a sexuality under the LGBTQIA+ umbrella have higher odds of reporting symptoms of clinical depression, frequent physical distress, and poor overall health compared to heterosexual people (Mansh et al., 2022). Similarly, acne may be particularly distressing for transgender women as it can interfere with their feminine gender expression (Gold et al., 2024) and is also associated with an increased likelihood of depression and anxiety in transmasculine people who have received testosterone (Braun, Zhang, et al., 2021). Research has also found that gay and bisexual men with alopecia experience greater appearance‐focused anxiety than heterosexual men (Zucchelli et al., 2024) and that gay men report feeling that their experiences are particularly difficult because of the emphasis on appearance in gay male culture (Zucchelli et al., 2022).

Research suggests that cancer‐related visible differences may also impact LGBTQIA+ people in unique ways. Some people with cancer report that visible differences such as scarring and having a stoma are a challenge to their LGBTQI^1^ identity and have a negative impact on their sexual embodiment, which makes them question their LGBTQI identity (Denes et al., 2025). However, research also indicates that some LGBTQIA+ people with cancer‐related visible differences may experience fewer challenges than cisgender/heterosexual people. For example, some research indicates lesbian women who undergo appearance‐altering treatment for breast cancer (e.g., mastectomy) report fewer appearance and sexual concerns, less thought avoidance and denial coping, and more use of support from friends and positive reframing than heterosexual women (Arena et al., 2007). This may be due to lesbian culture placing less importance on physical appearance (Alvy, 2013). This is supported by research involving queer women with cancer‐related scars and limb differences, who described queer communities as more accepting of their visible difference and felt that discovering their queer identity helped reduce appearance pressures they previously experienced (Ussher et al., 2023). Research relating to the impact of cancer‐related visible difference is mixed when it comes to the experiences of transgender people. In some cases, transgender people may find physical changes following cancer treatment gender affirming (Ussher, Power, et al., 2023). However, for others these changes can interrupt the gender affirmation process, contributing to or causing gender dysphoria (Taylor & Bryson, 2016).

As both LGBTQIA+ people and people with visible differences are stigmatised groups, LGBTQIA+ individuals with visible differences may experience an ‘intersectional stigma’ (i.e., the convergence of multiple stigmatised identities; Turan et al., 2019). Having intersecting marginalised identities can also result in additional barriers to care. For example, LGBTQIA+ people with chronic inflammatory skin diseases are more likely than cisgender/heterosexual people to report not being treated with respect by health care practitioners (Nock et al., 2023).

Although the above challenges related to visible differences are relevant across the lifespan, they may be especially pertinent for young people, as appearance is particularly salient during adolescence (Frisén et al., 2015). Additionally, many young people experience appearance‐related bullying, comments and teasing whether they have a visible difference or not (Lovegrove & Rumsey, 2005). As such, this life stage may be especially challenging for those with visible differences, with existing research with predominantly cisgender/heterosexual young people indicating that many experience elevated anxiety and depression (van Dalen et al., 2020) and difficulties with self‐esteem, body image, and quality of life (Siddiqui et al., 2024; Tiemens et al., 2013). Research also suggests LGBTQIA+ young people are at increased risk of body image concerns because of a combination of sociocultural and LGBTQIA+‐specific appearance pressures, ‘minority’ stressors, and developmental features (Roberts, 2024). Although existing research about LGBTQIA+ young people does not specifically include those with visible differences, this group is likely to be at even higher risk of negative outcomes, because of difficulties conforming to appearance standards and because having a visible difference is especially challenging during adolescence (Crerand et al., 2020). Finally, it may be more difficult for LGBTQIA+ young people with visible differences to cope with the challenges associated with looking different, because of the already elevated risk of poor mental health among LGBTQIA+ young people compared to cisgender/heterosexual young people (Becerra‐Culqui et al., 2018). Despite these risks, very little research exploring the experiences of LGBTQIA+ people with visible differences has exclusively focused on young people.

Overall, existing research on the experiences of LGBTQIA+ young people with visible differences is limited, as studies typically focus on either visible difference, LGBTQIA+ health, or LGBTQIA+ young people. When research does include LGBTQIA+ people with visible differences, this is typically limited to gay and bisexual men (e.g., Zucchelli et al., 2024), lesbian and bisexual women (e.g., Arena et al., 2007), or transgender people (e.g., Gold et al., 2024), excluding other LGBTQIA+ identities. Finally, current research is condition‐specific, limiting understanding of the experiences of LGBTQIA+ young people with rarer visible differences and commonalities in experiences across visible differences. Therefore, this research aimed to explore the lived experiences of LGBTQIA+ young people (aged 15–28 years) with a range of visible differences.

## METHODS

### Design

This study adopted an inductive qualitative design, using online qualitative surveys and reflexive thematic analysis (TA) to explore the lived experiences of LGBTQIA+ young people with visible differences. The research team consists of four White women; two identify under the LGBTQIA+ umbrella and three have experience of visible difference (one author is both LGBTQIA+ and has a visible difference). This research was given a favourable opinion by the University of the West of England's College Research Ethics Committee (reference CHSS.23.08.001) and participants gave informed consent.

### Data generation

An online qualitative survey was used to generate data. Qualitative surveys capture a diverse range of perspectives and experiences and are less intrusive than interviews, an important consideration when recruiting LGBTQIA+ young people who may not be ‘out’ to their family (Braun, Clarke, et al., 2021). The strong sense of ‘felt anonymity’ may also encourage participation by those uncomfortable interacting face‐to‐face with a researcher. Further, research suggests LGBTQIA+ young people are digitally active and that surveys can increase their engagement in research (McInroy, 2016).

Survey questions were developed based on previous qualitative research exploring the experiences of similar populations (e.g., Araya et al., 2021; Griffiths et al., 2012; Tran et al., 2020). An LGBTQIA+ young person with a visible difference and a researcher who has clinical and research experience with young people with visible differences also provided feedback on a draft survey. Key feedback included that the research was ‘really necessary’, terminology was appropriate, and the anonymity was a strength that would appeal to LGBTQIA+ people. Suggested optimisations included that the survey could be less formal, include more examples and explanations, and that some questions could be merged. This feedback was incorporated before recruitment began. The survey included questions about participants' experiences in LGBTQIA+ communities, experiences of their visible difference, and of intersections of LGBTQIA+ identity and visible difference. The survey was piloted through advertising it to a ‘subreddit’ for asexual people on Reddit; 21 responses were generated. After reviewing them, small amendments to the wording of some questions were made to clarify their meaning and elicit richer data. As the pilot data were in‐depth and no major changes were made to the survey, these data were included in the final dataset (for the pilot and final survey, see Supplementary Materials).

### Participants and recruitment

Young people aged 15–28 years who self‐identified as being LGBTQIA+ with a visible difference were eligible to take part. They were recruited in a variety of ways. The first and fourth authors work in partnership with a group of charitable organisations supporting people with visible differences, known as the Appearance Collective. The Appearance Collective organisations were asked to advertise the study on their websites, mailing lists, and social media pages. The study was also advertised by similar visible difference organisations outside the Appearance Collective, organisations supporting LGBTQIA+ people, via the Centre for Appearance Research's mailing lists, hard copy posters around the University of the West of England's campus, and through social media. Particular focus was given to recruiting a diverse participant group in terms of gender identity and sexuality, which was achieved through approaching online groups for underserved LGBTQIA+ communities. Participants were offered the opportunity to enter a prize draw for one £25 shopping voucher as a thank you for taking part. The demographics of the participant group were also reviewed throughout the data generation phase to determine which recruitment avenues to prioritise to recruit a diverse participant group.

There were 75 participants aged 15–27 years (*M* = 20.36). In line with guidance for conducting inclusive research (Malatino & Stoltzfus‐Brown, 2020), participants self‐described their gender, sexuality, and visible difference. Twenty‐four described their gender identity as (cisgender) female/woman, and 11 as (cisgender) male/man. A range of identities under the transgender umbrella were also represented (*n* = 26), and many participants described themselves using a combination of terms, such as ‘questioning/non‐binary/transmasculine’. Twenty‐five participants identified as asexual, 12 as bisexual, 9 as lesbian, 6 as gay, 2 as heterosexual and 10 as other identities under the LGBTQIA+ umbrella. Several participants described their sexuality using a combination of terms, such as ‘panromantic/demisexual’. A range of visible differences were also represented. Thirteen participants had more than one visible difference and over half (56%) identified as disabled, which for some was linked to their visible difference and for others it was not. Most participants (76%) described themselves as White and the remaining 24% self‐described as belonging to a range of racial/ethnic groups. The Supplementary Materials include further detail about participants' ages, gender identities, sexualities, visible differences, disabilities, racial/ethnic groups, and countries of residence.

### Data analysis

Reflexive TA was used to analyse the data and followed the six phases outlined by Braun and Clarke (2022). The first author led the analysis, with the other authors acting as ‘critical friends’ (Smith & McGannon, 2018, p. 113)—offering critical feedback on the developing interpretations and encouraging reflexivity. Although we did not anticipate imposter participants due to our targeted recruitment strategy and the very limited incentive offered, we were conscious of authenticity when reviewing the data. No responses stood out as suspicious. Our use of reflexive TA was underpinned by critical realism and contextualism, accessing reality through the lens of participants' lived experiences and perceptions and recognising that knowledge is impacted by sociocultural contexts (Maxwell, 2012). For further details of the analytic process see Supplementary Material. Braun and Clarke's (2025) Big Q Qualitative Reporting Guidelines (BQQRG), Reflexive Thematic Analysis Reporting Guidelines (RTARG; Braun & Clarke, 2024) and revised ‘checklist’ for good reflexive thematic analysis (Braun & Clarke, 2022) guided how this research was conducted and reported.

## RESULTS

One overarching theme and two themes were generated. Anonymised quotations from participants (P) are used to illustrate the analysis. Participants are identified with a number and their self‐reported demographics.

### Overarching theme: The weight of multiple marginalisation

Young people discussed the ways in which their LGBTQIA+ identity and visible difference made them feel different from others. They described how stigma relating to LGBTQIA+ identities and visible difference interacted and compounded their difficulties, although some experienced personal growth and felt their intersecting identities offered them unique perspectives. LGBTQIA+ communities provided a safe space for some participants that helped facilitate self‐acceptance, while others hid parts of their identity to avoid multiple discrimination. This overarching theme encompassed two themes: (1) *Dual identities*, *dual stigma* and (2) *Hiding to prevent hurting*.

### Theme 1: Dual identities, dual stigma

Young people worried about how others would perceive them due to their visible difference and LGBTQIA+ identity and wrote about the psychosocial consequences of having two (or more) marginalised identities. Having both a visible difference and being LGBTQIA+ was ‘*too much*’ (P55, aged 25, queer, transgender man, facial scars) compared to having one or the other. P31 and P70 both described the separation they felt from others and the negative impact on their mental health of not relating to cisgender/heterosexual people nor LGBTQIA+ people without visible differences:

When you're LGBTQIA+, you're already different from the cis/straight people. When you're queer with a visible difference, you're different from the queer people AND the cis/straight people. It can feel so isolating and alienating to be SO different. (P31, aged 21, bisexual, transgender man, eczema)

I think being part of the lgbtqia community can already sometimes have a negative impact so adding a visible difference to that almost makes you ‘double different’ which can just add to the feeling of isolation. (P70, aged 24, gay, male, pectus excavatum) 2

Young people also described the harm of experiencing both visible difference and LGBTQIA+ related discrimination. Both P73 and P42 discussed how LGBTQIA+ people with visible differences are especially susceptible to being targeted because of being different in multiple ways:

Being LGBTQIA+ and having a visible difference will result in a lot more challenges than being cishet with a visible difference as it will lead to being different in several ways, which will likely be an opportunity for bullying and discrimination. (P73, aged 18, asexual, female, amputated limb)

It's just one less thing to be bullied for in the case of heterosexual/cisgender people. The challenge is still present, of course, but they're not going to face the combined vulnerability of those with visible differences *and* those who are part of the LGBTQIA+ community. (P42, aged 18, bisexual/pansexual, transfeminine, ectodermal dysplasia) 3

Young people also described the double standards in how LGBTQIA+ young people with visible differences were treated compared to cisgender/heterosexual individuals, with the latter being perceived to be afforded more freedom to be visibly different because of their conformity to cisheteronormativity. Visible differences may be more acceptable for cisgender/heterosexual individuals as they only deviate from societal norms in one way. In comparison, those who are LGBTQIA+ challenge both appearance norms and dominant narratives positioning cisgenderism and heterosexuality as the default. P12 explained how these double standards show up:

It feels like cis/het people with visible differences are empowered and encouraged or celebrated for having those differences, but when someone is queer with those visible differences, to me it feels like they are frowned upon. (P12, aged 16, panromantic/demisexual, non‐binary, scars)

Relatedly, young people described how these double standards extended to health care. They felt that, despite experiencing similar visible differences, cisgender/heterosexual people received more favourable treatment. This was reported by P20, who felt that cisgender/heterosexual young people with visible differences may be more likely to receive greater psychosocial support, and by P67, who outlined the discrepancies in their care and the care of their cisgender/heterosexual friend with the same visible difference:

There's probably more support for them because they're the “norm.” (P20, aged 19, asexual, gender not disclosed, acanthosis nigricanis) 4

I have a friend who is cishet and has a cleft lip and palate like me that I met in elementary school. I've noticed that he gets better post‐surgery recovery care (like better timed follow‐ups). (P67, aged 17, asexual demipanromantic, non‐binary, cleft lip and palate)

It was clear the experiences of multiple marginalisation were especially damaging for transgender young people. P31 described an experience in which someone equated their ‘*disgusting*’ visible difference with their transgender identity, using it to reinforce the harmful idea that they were inherently flawed and unlovable. They described beginning to internalise these feelings over time:

People are cruel. Some already mistreat you just for being queer, add a visible difference to that? It's hell sometimes. I've had so many people tell me I'd be better off dead, that my skin is as disgusting as I am (for being trans) and I can never be loved. It's hard to learn to love yourself when people are telling you [that] you can't be loved. (P31, aged 21, bisexual, transgender man, eczema)

Similarly, P55, a transgender man, outlined how their life had become more complex since acquiring facial scars and described the pervasive social barriers that came with having a visible difference and being transgender. They described how navigating multiple layers of stigma took an extreme toll on their mental health that they did not feel equipped to cope with. The extent of this was such that, after acquiring their visible difference, leading a happy and fulfilling life was unimaginable and they had contemplated suicide:

Acquiring a cancer related facial disfigurement as a young trans person has led to a lot of isolation and barriers in everyday life. Everywhere there's gendered barriers, cancer stigma, faceism, and transphobia and dealing with the mix of them is exhausting. After my first cancer surgery when I acquired my facial disfigurement, I planned to take my life. I just couldn't imagine being a trans person with a disfigurement and living life. It already felt like whenever I went outside people were trying to figure me out ‐ my face and my gender and it was too much ‐ the stares and the questions. (P55 aged 25, queer, transgender man, facial scars)

Some young people found acceptance when spending time with other LGBTQIA+ people, which provided relief from the challenges they faced. They described various ways being around LGBTQIA+ people made them feel uplifted and attributed this to the accepting nature of LGBTQIA+ communities. P5 wrote about how this helped them feel their visible difference was socially acceptable:

I believe that the community being so accepting and supportive makes it easier to have a visible difference. There's not as much shame and the community makes it feel okay to have a difference. (P5, aged 18, biromantic asexual, female, scars)

LGBTQIA+ people's acceptance also helped young people positively appraise their situation, resulting in them feeling empowered and proud of their visible difference. This was highlighted by both P47 and P35:

I think lgbtqia+ people are accepting and understanding and empowering to people with visible differences. (P47, aged 27, bisexual, female, alopecia)

I think I feel better with myself because of that attitude of queer people. I feel proud of my visible differences. (P35, aged 18, asexual, transmasc enby, ileostomy and scars)

When it came to discussing their visible difference, young people therefore expressed a preference for doing so with other LGBTQIA+ people rather than cisgender/heterosexual people. They described feeling secure to do so, which was linked to the reduced likelihood of LGBTQIA+ people judging them. This was exemplified by P41 and P47, who shared their increased willingness to be open about their experiences of their visible difference with other LGBTQIA+ people:

I feel much safer to talk about my experiences with my condition with LGBT people, as they are much less likely to judge someone's appearance. (P41, aged 23, bisexual, non‐binary/transmasculine, facial difference)

I don't spend a lot of time in lgbtqia+ spaces but when I do I feel welcome and as though I can be myself. I would feel more comfortable opening up to someone about the nature of my visible difference in these kinds of safe spaces. (P47, aged 27, bisexual, female, alopecia)

Although many young people wrote about challenges associated with having multiple marginalised identities, some described the positive ways they viewed their differences and the unique perspectives and opportunities they provided them. P32 felt that LGBTQIA+ young people with visible differences were more likely to understand the degree of diversity within LGBTQIA+ populations than those without visible differences:

I think that they [LGBTQIA+ young people with visible differences] have a more complete picture of what the queer community is like and are more likely to understand intersectionality. (P32, aged 21, lesbian, femme, ostomy bag and wheelchair/cane)

Several young people also felt that their experiences had improved their ability to empathise with, and be accepting of, others. For example, both P74 and P54 described the personal growth that resulted from their intersecting identities with a sense of pride and positivity:

I am accepting of literally everything. Like my experiences have made me so open to difference. (P74, aged 19, queer, transmasc/non‐binary, eczema and scars)

Our experiences are rich and unique. I love intersecting multiple identities and I feel it has overall made me a kinder, more accepting, more loving, and more compassionate person than if I only identified with one or none. (P54, aged 24, bisexual/pansexual, non‐binary agender, cleft lip and palate)

Overall, being LGBTQIA+ and having a visible difference had a complex and intersectional impact on young people, resulting in poor treatment from others and negatively affecting their wellbeing. However, some felt they were accepted by other LGBTQIA+ people, which helped them feel more positive towards their visible difference and improved their wellbeing. Additionally, some young people experienced personal growth due to their intersecting identities.

### Theme 2: Hiding to prevent hurting

Some young people described being fearful of the social consequences of having multiple marginalised identities. As they already felt marginalised in relation to their visible difference, they hid their LGBTQIA+ identity to avoid further negative treatment. Others' reactions towards their visible difference were therefore a barrier to coming out. This was highlighted by P58, who described not wanting to come out to avoid being made to feel any more different than they already did, and P65, who felt that it was easier for LGBTQIA+ people without visible differences to come out:

I think it [my visible difference] makes me less inclined to share my [LGBTQIA+] identity as it adds yet another “other” to me. (P58, aged 24, bisexual, woman, neurofibromatosis) 5

I imagine it's easier to come out [for LGBTQIA+ people without visible differences]. After all you aren't adding another label to your already label‐filled personhood if you aren't visibly disabled. (P65, aged 21, biromantic, female, eczema)

Young people also seemed to have internalised stigma towards their visible differences and LGBTQIA+ identity. This resulted in them wanting to hide their identities, as described by P63, who wrote about the shame they felt relating to their LGBTQIA+ identity and visible difference:

It has made it harder to come out as I feel a sense of shame having a different physical appearance and sexuality. (P63, aged 24, bisexual, woman, eczema)

Negative reactions from others after coming out also had the potential to affect various aspects of young people's everyday lives. For example, P34 described their parents' concerns that being open about their LGBTQIA+ identity could cost them not only their current job but threaten their future employment, which were already at risk due to their visible difference:

I've been told by my parents that I cannot come out to anyone because my visible differences make me a target at work and that I would be unemployable were I to be open about another marginalized identity. (P34, aged 21, lesbian/homoflexible, gender non‐conforming, scars and wheelchair/cane)

For some, fear of the consequences of disclosure related to both their LGBTQIA+ identity and their needs relating to their visible difference. P8 feared that disclosing their LGBTQIA+ identity would result in being judged and marginalised even more than they already were and felt disclosing their desire to use a mobility aid would result in a similar outcome. As with other young people, the risks associated with disclosing were not viewed as being worth it:

I worry about judgement frequently, to the point that I haven't come out yet or even expressed my desire to have a mobility aid. I already know I'm part of a marginalised group and I just don't want to risk receiving more hate and judgement. (P8, aged 19, asexual demiromantic, transmasc lavender boy, burn scars)

In sum, this theme reflected young people's tendency to conceal their LGBTQIA+ identity as a way of avoiding multiple marginalisation.

## DISCUSSION

This study explored the lived experiences of LGBTQIA+ young people with visible differences. Young people highlighted the psychosocial challenges of having intersecting marginalised identities, which included negative feelings about themselves and increased risk of poor treatment from others. Many discussed their experiences of multiple discrimination. This echoes previous research with other marginalised groups with visible differences, such as people of the global majority (e.g., Costa, 2022; Thompson et al., 2010), as well as research with LGBTQIA+ people with other intersecting marginalised identities, such as LGBTQIA+ disabled people (e.g., Kempapidis et al., 2024). It highlights the need to adopt an intersectional lens when exploring the experiences of individuals from such groups. This is especially important given that stigma and discrimination faced by marginalised groups have been shown to increase stress and negatively impact both physical and mental health, as explained by the Minority Stress Model (Brooks, 1981). Additionally, research suggests multiple discrimination can negatively impact health care utilisation, quality of care, and outcomes relating to education, employment, finance, and romantic relationships, therefore substantially impacting individuals' lives (Agénor et al., 2022; Kempapidis et al., 2024).

Young people also highlighted perceived inequalities in treatment between LGBTQIA+ young people with visible differences and their cisgender/heterosexual counterparts, including in health care. This is supported by research indicating some LGBTQIA+ people experience discrimination in health care settings, including denial or refusal of care and verbal and physical abuse (e.g., Ayhan et al., 2019; Gioia & Rosenberger, 2022). Negative experiences may be further exacerbated by multiple marginalised identities, as research suggests health care professionals may struggle to consider intersecting identities and instead focus upon one in isolation (Semlyen et al., 2018).

Both cisgender and particularly transgender participants felt challenges were more significant for transgender young people. This echoes research indicating that transgender people face unique challenges in health care settings, including ‘trans broken arm syndrome’, where health care professionals incorrectly assume that their health condition is caused by their gender identity or gender‐affirming care (Wall et al., 2023). It is also supported by results from a systematic review of LGBT health inequities that found transgender women face unique forms of discrimination that negatively impact their health outcomes (Medina‐Martínez et al., 2021). The authors specifically highlighted the impact of transmisogyny, which occurs when transphobia and misogyny intertwine (Serano, 2016). This supports the current study by exemplifying the additional challenges faced by transgender people.

Despite the challenges that young people faced, some felt accepted by other LGBTQIA+ people. This helped them feel safer, more positive about their visible difference and more comfortable discussing it. This echoes research that suggests spending time in LGBTQIA+ social spaces may be protective. For example, some research indicates attending gay pride events can promote social connectedness and protect against the harms of discrimination (Schimanski & Treharne, 2019), and greater presence of LGBQ peers at school can protect against emotional distress (Eisenberg et al., 2016). Additionally, it reflects research on queer women with cancer‐related scars and limb differences who reported their visible differences were more accepted by queer communities (Ussher et al., 2023). This aligns with the stress‐buffering hypothesis (Cohen & Wills, 1985), which theorises that social support can positively impact people's health in the face of stressful situations by protecting against the harmful impact of stress (e.g., minority stress caused by discrimination). In the current study, relationships with other LGBTQIA+ people were likely an important source of social support for young people to buffer against the discrimination they experienced in everyday life, helping to explain their increased feelings of safety among LGBTQIA+ people and the alleviation of some of their negative feelings.

Some young people in the current study described concealing their LGBTQIA+ identity. Existing literature suggests that those with multiple marginalised identities face continual decisions about how open they are willing to be about their identities and may carefully consider how they disclose them, and how to avoid discrimination and vilification (Garvey et al., 2019; Perlson et al., 2021). They may also stay silent about one or more of their identities to avoid negative social consequences; however, this is complicated because many identities cannot be concealed (McNair, 2017). Indeed, some visible differences cannot easily be concealed (e.g., facial differences), which may explain why many young people chose to instead conceal their LGBTQIA+ identities.

Contrary to the challenges described above, some young people viewed their intersecting identities as improving their lives by increasing their ability to be accepting of and empathise with others. This may be because these young people had learnt to cope with the physical and psychosocial challenges of their visible difference. While factors contributing to adjustment in people with visible differences are not yet well understood (Guest et al., 2024), the current study echoes previous research suggesting some young people experience personal growth as a result of their visible difference, including increased appreciation and enjoyment of life, assertiveness and self‐confidence in interactions with others, and greater appreciation for personal qualities that are not associated with appearance (Wallace et al., 2007; Williamson et al., 2010). Contrarily, appearance concerns have been shown to be one of the most significant barriers to adjustment (Gee et al., 2020; Stock et al., 2020). Varying levels of appearance concerns may therefore help explain the differences in young people's appraisal of their intersecting identities in the current study.

### Implications

Multilevel interventions are needed to focus on supporting LGBTQIA+ young people with visible differences and promoting societal‐level change. This is especially the case given many challenges faced by these populations result from others' negative perceptions and reactions.

Societal‐level interventions should focus on reducing discrimination against LGBTQIA+ people and those with visible differences. Some existing interventions have separately addressed stigma towards LGBTQIA+ people and people with visible differences and indicated positive results, including educational campaigns and intergroup contact (e.g., Topp et al., 2019; Tran et al. 2022). However, it is also important to consider ways to target the multiple discrimination encountered by LGBTQIA+ young people with visible differences. This could involve tailoring the above interventions to be focused specifically on LGBTQIA+ young people with visible differences, for example by providing education about intersectionality and related challenges. Similarly, increased contact between members of the general population and LGBTQIA+ people with visible differences could help reduce multiple discrimination. Future research could investigate the effectiveness of these interventions.

The current study indicates the need for interventions focused on improving health care professionals' perceptions and knowledge about how to effectively support LGBTQIA+ young people with visible differences. LGBTQIA+ people report needing health care that is tailored to their needs (Waters et al., 2024), and research suggests improving health care professionals' empathy and understanding of LGBTQIA+ people's experiences can enable them to provide such care (Boos et al., 2019). Additionally, previous research has identified several aspects of inclusive care of LGBTQIA+ young people related to health care professionals. A review by Laiti et al. (2019) indicated that inclusive health care professionals use inclusive language, understand relevant issues for LGBTQIA+ young people's health and wellbeing, have knowledge about diversity in gender identity and sexuality, and are sensitive in their interactions with LGBTQIA+ young people. As such, training could be developed to educate health care professionals about the unique needs of LGBTQIA+ young people with visible differences and how to be more inclusive practitioners. When doing so, the perspectives of LGBTQIA+ young people with visible differences should be incorporated to help ensure training is relevant to their needs. These interventions are particularly important given that many people with visible differences are in regular contact with health care professionals.

This study also highlights the need for interventions to support LGBTQIA+ young people with visible differences to cope with the challenges they face. Although psychosocial interventions have been used to support young people with visible differences (see Jenkinson et al., 2015; Waite et al., 2024), LGBTQIA+ young people with visible differences appear to have unique needs that have not yet been considered. Peer support may be one effective avenue for tailored interventions, given that the participants in the current study described the positive impact of spending time with other LGBTQIA+ people. Evidence indicates peer support can help by combating isolation, boosting self‐esteem and confidence, reducing the likelihood of risk‐taking behaviours, and helping individuals overcome the challenges of having multiple marginalised identities (Borthwick et al., 2020). Therefore, it could be helpful for LGBTQIA+ young people with visible differences to find communities where their identities are valued and represented.

Existing visible difference support organisations and communities could also improve inclusivity of LGBTQIA+ people in a number of ways. This includes recognising how the additional challenges faced by LGBTQIA+ people shape their support needs and being mindful of the language and assumptions embedded within their work. For example, taking care to avoid binary terminology and presuming an individual's gender identity, sexuality, or desire for sexual or romantic relationships. In addition, efforts should be made to increase representation of LGBTQIA+ people by using diverse imagery and examples, for example on organisations' websites. Including LGBTQIA+ people in the design and delivery of services and resources can also help ensure that they feel represented and that the support has considered their needs. Finally, organisations should consider that LGBTQIA+ people with visible differences may experience additional barriers when it comes to seeking support and may have fewer resources available to cope with challenges compared to cisgender/heterosexual people. This may mean they require a greater amount of support to engage with resources and that professionals need to take extra time to build trust and create safe, affirming spaces. As such, organisations should account for this in their service planning to help promote equitable access to support.

### Methodological considerations

This study had a number of strengths. Firstly, the participant group was large and diverse in terms of sexualities, gender identities, and visible differences. This helped address limitations of previous qualitative research on LGBTQIA+ identities and visible difference, which has been condition‐specific and focused on a small range of LGBTQIA+ identities. This is important in furthering our understanding of the experiences and needs of those who are underrepresented in LGBTQIA+ health research, such as genderqueer people (Smalley et al., 2016), as well as in visible difference research, such as those with rarer conditions. Relatedly, most young people in the current study had never received psychosocial support for their visible difference. This is a notable difference from most participants in visible difference research, who are often recruited through organisations supporting people with visible differences and have therefore typically received psychosocial support. Reaching those who have not received support is important as they may have different psychosocial experiences. It also helps ensure resources that are developed are appropriate for the needs of a range of individuals.

There were also limitations to this study. The majority (76%) of participants identified as White. This may be in part because the research team's positionality was disclosed on recruitment materials, including information about the team all being White. People of the global majority may therefore not have felt comfortable to take part. Although steps were taken to address this, such as recruiting in spaces for people of the global majority and explicitly stating the research welcomed those with intersecting identities, it likely contributed to their lack of representation in the participant group. Further research is needed to better understand these young people's experiences. This is especially important given that embodied experiences differ between racial/ethnic groups (e.g., the unique role of hair and hairstyles in Black people's identity; Lashley, 2020). Additionally, although recruiting internationally contributed to the size and diversity of the participant group, it limits the extent to which the analysis can be used to inform intervention development tailored to specific cultural and health care settings. Future research is therefore needed that focuses on people from specific cultural groups or countries to ensure that interventions are tailored to specific needs and settings.

Despite the use of an international participant group, most participants lived in Western countries and therefore their experiences will have been shaped by Western norms around gender, sexuality, and appearance, which differ substantially from those in other cultures. In particular, gender is often framed as a binary in the West (Boero & Mason, 2020). Conversely, people with suprabinary genders not only exist but are accepted in many non‐Western and Indigenous cultures (e.g., Lim‐Bunnin, 2020; Wei et al., 2024). As such, the experiences of LGBTQIA+ young people with visible differences living in these cultures are likely to be significantly different from those in the current study, warranting additional research.

Finally, the participant consultation in this study was limited because of the unfunded, time‐constrained nature of the research. While this was partially addressed by including space for participant feedback in the survey, future research should incorporate more substantial participant involvement.

## CONCLUSION

This study provides initial, rich insight into the lived experiences of LGBTQIA+ young people with visible differences. It presents a novel contribution by exploring the under‐researched intersection between visible difference and LGBTQIA+ identities, using a qualitative and cross‐condition approach, and focusing on young people with a diverse range of LGBTQIA+ identities. It suggests that LGBTQIA+ young people with visible differences have unique needs that are unlikely to be met by generic support and instead require tailored intervention. Future research is needed to co‐produce interventions that can improve the lives of LGBTQIA+ young people with visible differences and help reduce inequities.

## AUTHOR CONTRIBUTIONS

**Emma Waite:** Conceptualization; methodology; formal analysis; investigation; project administration; visualization; writing – original draft. **Elizabeth Jenkinson:** Conceptualization; methodology; supervision; writing – review and editing. **Victoria Clarke:** Conceptualization; methodology; supervision; writing – review and editing. **Ella Guest:** Conceptualization; methodology; supervision; writing – review and editing.

## CONFLICT OF INTEREST STATEMENT

The authors declared no potential conflicts of interest with respect to the research, authorship, and/or publication of this article.

## ETHICAL APPROVAL

This research was given ethical approval by the University of the West of England's College Research Ethics Committee (reference number CHSS.23.08.001).

## Supporting information


**Supplementary File A.** Pilot and final survey.
**Supplementary File B**. Detailed demographic information.
**Supplementary Table 1**. Additional demographic information.
**Supplementary File C**. Additional detail about analytic process.

## Data Availability

Research data are not shared.

## Footnotes

^1^
Changes in acronyms indicate the specific LGBTQIA+ populations included in each study.

^2^
A condition that causes the breastbone to sink into the chest.

^3^
A condition that may cause missing, pointed, globe‐shaped, or small teeth, a large forehead and/or decreased skin pigment.

^4^
A skin condition characterized by dry, velvety, dark patches of skin.

^5^
A condition that can cause café au lait spots and/or tumours.
