# Supplementary material for: Exploring the lived experiences of LGBTQIA+ young people with visible differences: Being ‘double different’
Source: Br J Health Psychol. 2026 Nov 4;31(4):e70118. doi: 10.1111/bjhp.70118 (PMC13732410; doi:10.1111/bjhp.70118)
Supplement: Supplementary file 1 [file BJHP-31-0-s001.docx]

**Supplementary File A – Pilot and Final Survey**

**Pilot Survey**

**Some questions about you**

In order for me to learn about the range of people taking part in this research, and to describe the broad characteristics of my participant group in my report, I would be grateful if you could answer the following questions. Please write your answer in the space provided. All these questions are optional – if you prefer not to answer a question, please leave the space blank. Please note that the information provided by all participants will be aggregated.

How old are you? (open text box)

How would you describe your gender identity? (open text box)

Employment – tick all that apply: FT employed, PT employed, unemployed, FT student, PT student, something else (please describe):

- If you work, what is your occupation?

How would you describe your sexuality? (open text box)

How would you describe your social class? (open text box)

What is your racial/ethnic background? (open text box)

Do you consider yourself to be disabled? Yes/no

- If yes, if you're happy to do so, please describe the nature of your disability:

What is the nature of your visible difference? (e.g., scar, birthmark, skin condition such as eczema or psoriasis, amputated limb, craniofacial condition such as cleft lip and/or palate) (open text box)

Were you born with your visible difference? Yes/no

- If no, how old were you when you acquired your visible difference? (open text box)
- If no, how did you acquire your visible difference? (open text box)

**Main Survey Questions**

**Visible Difference**

The following questions relate to your experiences as a young person with a visible difference as well as your thoughts and feelings about these. There are no right or wrong answers; we are interested in hearing your perspective so please write as much or as little as you would like. If you would prefer not to answer a question, please leave the space blank.

Please describe your visible difference and how it affects your appearance:

Do you feel that your visible difference impacts your everyday life? If yes, please describe how it impacts your life and your thoughts and feelings about this:

**LGBTQIA+ Identity**

The following questions relate to your experiences as an LGBTQIA+ young person as well as your thoughts and feelings about these. There are no right or wrong answers; we are interested in hearing your perspective so please write as much or as little as you would like. If you would prefer not to answer a question, please leave the space blank.

Are you currently ‘out’ (i.e., have told others that you are part of the LGBTQIA+ community)?

Do you have any close relationships with other LGBTQIA+ people (e.g., family, friends)? If yes, please briefly describe the relationship(s):

Do you spend time in LGBTQIA+ social spaces? If yes, please describe the LGBTQIA+ social spaces where you spend time:

**LGBTQIA+ Identity + Visible Difference**

The following questions relate to your experiences as an LGBTQIA+ young person with a visible difference as well as your thoughts and feelings about these. There are no right or wrong answers; we are interested in hearing your perspective so please write as much or as little as you would like. If you would prefer not to answer a question, please leave the space blank.

What do you think the attitude is of the LGBTQIA+ community towards physical appearance?

- How do these attitudes impact you as an LGBTQIA+ young person with a visible difference?

In what ways do you think your visible difference has impacted and/or currently impacts on your experiences as an LGBTQIA+ young person?

Are there any positive aspects to being a member of the LGBTQIA+ community who has a visible difference? If yes, please describe these positive aspects as well as your thoughts and feelings about them.

Have you ever experienced any challenges due to being a member of the LGBTQIA+ community who has a visible difference? If yes, please describe these challenges as well as your thoughts and feelings about them.

In what ways do you think the experiences of LGBTQIA+ young people with visible differences differ from LGBTQIA+ young people without visible differences?

In what ways do you think the experiences of heterosexual and cisgender young people with visible differences differ from those who are LGBTQIA+ with similar visible differences?

Have you ever received any psychological or social support related to your visible difference? (Yes/no)

Have you ever received any psychological or social support related to your visible difference? (Yes/no)

Have you ever received any psychological or social support related to your identity as an LGBTQIA+ person? (Yes/no)

- If yes to either: Please describe the support you have received.

What do you think are the main areas that LGBTQIA+ young people with visible differences may need support with?

**Other**

Is there anything else you would like to add about your experiences as an LGBTQIA+ young person with a visible difference?

How could we improve this survey? (e.g., is there anything we’ve missed?, anything we should remove?, is the language appropriate?)

We may do some follow-up interviews to find out more after this survey. Are there any particular questions we should include in these interviews? Or are there any topics that you think we should ask more about?

Sometimes in research interviews we ask people to use photographs to help tell stories or represent their experiences. As such, there may be an option to show personal photographs in these interviews as a way to facilitate discussion. Do you have any thoughts about the usefulness of this option? Are there any aspects you think the photos should focus on?

Would you be happy to be contacted about future research? If yes, you will be asked to provide a contact email address. (Yes/no)

As a thank you for taking part in this research, you are invited to enter into a prize draw. One person who enters will receive a £25 shopping voucher. Would you like to enter the prize draw? If yes, you will be asked to provide a contact email address.

Please follow this link to provide an email address we can use to contact you. This will be stored separately from your survey response:

**Final Survey (changes indicated in red)**

**Some questions about you**

In order for me to learn about the range of people taking part in this research, and to describe the broad characteristics of my participant group in my report, I would be grateful if you could answer the following questions. Please write your answer in the space provided. All these questions are optional – if you prefer not to answer a question, please leave the space blank. Please note that the information provided by all participants will be aggregated.

How old are you? (open text box)

What country do you live in? (open text box)

How would you describe your gender identity? (open text box)

Employment – tick all that apply: FT employed, PT employed, unemployed, FT student, PT student, something else (please describe)

- If you work, what is your occupation?

How would you describe your sexuality? (open text box)

How would you describe your social class/socioeconomic status? (open text box)

What is your racial/ethnic background? (open text box)

How would you describe your religious/spiritual beliefs? (e.g., Atheist, Taoist, Christian, Buddhist)

Do you consider yourself to be disabled? Yes/no

- If yes: If you're happy to do so, please describe the nature of your disability:

What is the nature and severity of your visible difference? (e.g., scar, birthmark, skin condition such as eczema or psoriasis, amputated limb, craniofacial condition such as cleft lip and/or palate). (open text box)

Were you born with your visible difference? Yes/no

- If no: How old were you when you acquired your visible difference? (open text box)
- If no: How did you acquire your visible difference? (open text box)

**Main Survey Questions**

**Visible Difference**

The following questions relate to your experiences as a young person with a visible difference as well as your thoughts and feelings about these. There are no right or wrong answers; we are interested in hearing your perspective so please write as much or as little as you would like. If you would prefer not to answer a question, please leave the space blank.

How do you feel your visible difference affects your appearance? (Updated from ‘Please describe your visible difference and how it affects your appearance’)

Do you feel that your visible difference impacts your everyday life? If yes, please describe how it impacts your life and your thoughts and feelings about this:

**LGBTQIA+ Identity**

The following questions relate to your experiences as an LGBTQIA+ young person as well as your thoughts and feelings about these. There are no right or wrong answers; we are interested in hearing your perspective so please write as much or as little as you would like. If you would prefer not to answer a question, please leave the space blank.

Are you currently ‘out’ (i.e., have told others that you are part of the LGBTQIA+ community)?

Do you have any close relationships with other LGBTQIA+ people (e.g., family, friends)? If yes, please briefly describe the relationship(s):

Do you spend time in LGBTQIA+ social spaces? If yes, please describe the LGBTQIA+ social spaces where you spend time:

**LGBTQIA+ Identity + Visible Difference**

The following questions relate to your experiences as an LGBTQIA+ young person with a visible difference as well as your thoughts and feelings about these. There are no right or wrong answers; we are interested in hearing your perspective so please write as much or as little as you would like. If you would prefer not to answer a question, please leave the space blank.

What do you think is the attitude of the LGBTQIA+ community towards physical appearance?

- How do these attitudes impact you as an LGBTQIA+ young person with a visible difference?

In what ways do you think your visible difference has impacted and/or currently impacts on your experiences as an LGBTQIA+ young person?

Are there any positive aspects to being a member of the LGBTQIA+ community who has a visible difference? If yes, please describe these positive aspects as well as your thoughts and feelings about them:

Have you ever experienced any challenges due to being a member of the LGBTQIA+ community who has a visible difference? If yes, please describe these challenges as well as your thoughts and feelings about them:

In what ways do you think the experiences of LGBTQIA+ young people with visible differences differ from LGBTQIA+ young people without visible differences?

In what ways do you think the experiences of heterosexual and cisgender young people with visible differences differ from those who are LGBTQIA+ with similar visible differences?

Have you ever received any professional (updated from ‘psychological or social’) support related to your visible difference? (e.g., therapy, peer support group) (Yes/no)

Have you ever received any professional (updated from ‘psychological or social’) support related to your identity as an LGBTQIA+ person? (e.g., therapy, peer support group) (Yes/no)

- If yes: Please describe the support you have received:

What do you think are the main areas that LGBTQIA+ young people with visible differences may need support with?

**Other**

Is there anything else you would like to add about your experiences as an LGBTQIA+ young person with a visible difference?

How did you hear about this study?

How could we improve this survey? (e.g., is there anything we’ve missed?, anything we should remove?, is the language appropriate?)

We may do some follow-up interviews to find out more after this survey. Are there any particular questions we should include in these interviews? Or are there any topics that you think we should ask more about?

Sometimes in research interviews we ask people to use photographs to help tell stories or represent their experiences. As such, there may be an option to show personal photographs in these interviews as a way to facilitate discussion. Do you have any thoughts about the usefulness of this option? Are there any aspects you think the photos should focus on?

Would you be happy to be contacted about future research? If yes, you will be asked to provide a contact email address. (Yes/no)

As a thank you for taking part in this research, you are invited to enter into a prize draw. One person who enters will receive a £25 shopping voucher. Would you like to enter the prize draw? If yes, you will be asked to provide a contact email address.

Please follow this link to provide an email address we can use to contact you. This will be stored separately from your survey response:

**Supplementary File B – Detailed Demographic Information**

Twenty-four participants described their gender identity as either cisgender female, cisgender woman, female, or woman, and 11 self-described as either male, cisgender male, cisgender man, or man. A range of identities under the transgender umbrella were also represented, including participants who self-described using the terms transgender male or transgender man (*n* = 4), transgender woman (*n* = 4), transmasculine non-binary (*n* = 3), transsexual man (*n* = 1), transfeminine (*n* = 1), transmasculine (*n* = 1), transmasculine lavender boy (*n* = 1), genderqueer transmasculine (*n* = 1), and “somewhere under the trans non-binary umbrella” (*n* = 1). Further gender identities included non-binary (*n* = 5), agender (*n* = 2), gender non-conforming (*n* = 1), and genderfluid (*n* = 1). Finally, many participants described themselves using a combination of terms. Examples included “questioning/non-binary/transmasculine”, “non-binary/gender non-conforming/genderqueer”, “non-binary/agender”, “transmasculine, non-binary, agender and xenogender”, and “fluctuating but mostly female presenting”.

When asked to describe their sexuality, 25 participants labelled themselves as asexual, including those that identified as asexual biromantic, asexual panromantic, aromantic asexual, asexual demipanromantic, and asexual demiromantic. Twelve participants identified as bisexual, 9 as lesbian (including 1 as lesbian/homoflexible), and 6 as gay. Further sexualities included pansexual (*n* = 4), queer (*n* = 4), heterosexual (*n* = 2, both transgender), demisexual (*n* = 1), and biromantic (*n* = 1). Several participants described their sexuality using a combination of terms, including “pansexual/asexual”, “panromantic/demisexual”, “bisexual/pansexual”, “queer T4T”, “queer bisexual”, “asexual lesbian”, “straight (male preference)”, “gay, fictosexual, aegosexual, nindivian”, and “attracted to men and non-binary people”.

A range of visible differences were represented in the participant group. These included conditions such as acne, alopecia, birthmarks, cleft lip and/or palate, eczema, ectodermal dysplasia (a condition that may cause missing, pointed, globe-shaped or small teeth, a large forehead, and/or decreased skin pigment), limb differences, and neurofibromatosis (a condition that can cause café au lait spots and/or tumours), as well as acquired visible differences such as hyperpigmentation, limb amputation, and scars, and those caused by ostomies and relating to the use of mobility aids. Thirteen participants described having more than one visible difference. Over half (56%) identified as disabled. Disabilities (aside from visible differences) as described by participants included mental health conditions (anxiety, borderline personality disorder, depression, complex post-traumatic stress disorder, obsessive-compulsive disorder), sensory impairments (profound deafness), physical health conditions (endometriosis, spinal muscular atrophy, Sjögren’s syndrome, postural orthostatic tachycardia syndrome, Crohn’s disease, gastroparesis, Fowler’s syndrome, fibromyalgia, chronic fatigue syndrome, neuropathy), various types of neurodivergence (attention deficit hyperactivity disorder, autism, dyspraxia), and functional neurological disorder.

Table 1 provides additional demographic information about the participant group, including age, country of residence, and racial/ethnic background. The question about country of residence was added as a result of piloting and participant feedback and so this information is not available for the 21 pilot participants.

**Table 1**

*Additional Demographic Information*

| Demographic |  | N |
| --- | --- | --- |
| Age | 15 | 1 |
|  | 16 | 5 |
|  | 17 | 6 |
|  | 18 | 12 |
|  | 19 | 10 |
|  | 20 | 6 |
|  | 21 | 8 |
|  | 22 | 6 |
|  | 23 | 7 |
|  | 24 | 11 |
|  | 25 | 1 |
|  | 27 | 2 |
| Country | Australia | 2 |
|  | Austria | 1 |
|  | Belgium | 1 |
|  | Brazil | 1 |
|  | Canada | 4 |
|  | Croatia | 1 |
|  | Italy | 1 |
|  | Poland | 1 |
|  | South Africa | 1 |
|  | Sweden | 1 |
|  | UK | 21 |
|  | US | 18 |
|  | Not asked | 21 |
|  | Did not answer | 1 |
| Racial/ethnic background | African American | 1 |
|  | Biracial | 1 |
|  | British | 1 |
|  | Chinese | 1 |
|  | European and Jewish | 1 |
|  | Hispanic Mexican | 1 |
|  | Latino | 2 |
|  | Middle Eastern Egyptian | 1 |
|  | Mixed race | 2 |
|  | Scandinavian and Persian | 1 |
|  | South African and British | 1 |
|  | Taiwanese | 1 |
|  | White | 50 |
|  | White/Maori | 1 |
|  | White/Middle Eastern | 1 |
|  | White British | 1 |
|  | White European | 5 |
|  | White European/South American | 1 |
|  | White Irish | 1 |
|  | Did not answer | 1 |
|  |  |  |

**Supplementary File C – Additional Detail about Analytic Process**

**Data Analysis**

To familiarise herself with the data, the first author read and re-read the survey responses. She made notes about patterns that she noticed as well as her reflections on participants’ responses. She also discussed her reflections with the second, third, and fourth authors. The first author then coded the entire dataset by creating a table with two columns on Microsoft Word; one column to display the data and the other the corresponding codes. She used an inductive approach, being led by the data rather than analysing it through the lens of pre-existing theory (Braun & Clarke, 2021). She engaged in a close reading of the data to generate codes, both semantic and latent; however, gave particular attention to coding semantically to stay close to the meaning expressed by participants. She completed two rounds of coding; the first by question and the second by participant, starting at participant 50, then completing 51-75, 1-48, and finishing with participant 49. Changing the coding order in this way can be beneficial as it helps avoid later coded items receiving more insight than those coded earlier, may disrupt the familiar ‘flow’ for the dataset, and allows a different perspective on the data (Braun & Clarke, 2022).

Following coding, the first author grouped codes into clusters to generate candidate themes. Candidate themes were developed further and checked against the survey data to help ensure they meaningfully represented participants’ experiences. As part of this process, a thematic table and map were used, and several codes and candidate themes were moved and discarded. The first author then refined the resulting themes by ensuring they each had a clear central organising concept and were distinct from one another. She decided upon the final theme names and wrote detailed descriptions of each. Finally, in the writing up phase she expanded on these descriptions and selected quotes to illustrate the points made, adding further interpretation of these throughout.

**Quality Practices**

Braun and Clarke (2022) outline strategies that can be adopted by researchers to promote quality reflexive thematic analysis. In line with these, the first author engaged in reflexive journalling throughout the analytic process, for example, noting down her thoughts and reactions to particular data items and questioning the origins of these. She also kept an audit trail throughout the analytic process, consisting of lists of codes and all iterations of thematic tables and themes as they were developed from candidate into final themes. Finally, she discussed the analysis with her supervision team at various points and gained their perspectives. This input encouraged reflexivity and helped deepen her insight and interpretation (Smith & McGannon, 2018).
